# Desflurane Versus Opioid Anesthesia for Cardiac Shunt Procedures in Infants With Cyantoic Congential Heart Disease

**DOI:** 10.5812/aapm.9511

**Published:** 2013-07-01

**Authors:** Poonam Malhotra, George Mychaskiw, Amit Rai

**Affiliations:** 1 India Institute for Medical Sciences, New Delhi, India; 2 Nemours Children’s Hospital, Orlando, USA

**Keywords:** Research, Humans, Anesthesia, Heart Diseases, Desflurane, Analgesics, Opioid

## Abstract

**Background:**

Placement of a Blalock-Taussig (BT) shunt is frequently performed for palliation of cyanotic congenital heart disease (CCHD).

**Objectives:**

Inhalational anesthetics, when used in adult heart surgery, offer advantages of myocardial protection and decrease in use of inotropes, duration of ventilation, ICU and hospital length of stay (LOS).There is little literature, however, in the comparative use of inhalational and narcotic anesthesia in CCHD.

**Patients and Methods:**

Following Institutional Ethical Review Board approval and parental consent, 35 patients presenting for BT shunt were prospectively randomized to receive either a desflurane anesthetic or a narcotic anesthetic. Institutional practice for all patients undergoing BT shunt is to undergo cardiopulmonary bypass (CPB) following median sternotomy. Induction was accomplished with 5-7% sevoflurane in 100% oxygen, 2ug/kg fentanyl, 0.05mg/kg midazolam and 0.1mg/kg vecuronium. After intubation, patients in the narcotic group (n=16) received an additional 5-10ug/kg fentanyl, 0.1mg/kg midazolam, 100% oxygen and vecuronium. Patients in the inhalational group (n=19) received desflurane, 0.6-1 MAC, 100% oxygen, 0.05mg/kg midazolam, IV paracetamol 15mg/kg and vecuronium. At the end of surgery, patients were transferred to the ICU and received IV paracetamol and midazolam. Ventilation was weaned when the patient was hemodynamically stable. Demographics, baseline, intra and post-op heart rates, duration of inotrope use, ICU and hospital LOS, pre and post-op creatinine and serious adverse events (SAE) were recorded. Data were analyzed using Student, paired t, Mann-Whitney U and Chi square/Fisher exact tests, P < 0.05 significant.

**Results:**

Demographic data were similar, except for a modestly higher pre-op heart rate in the group receiving opioid anesthesia. Patients receiving desflurane had a significantly shorter duration of mechanical ventilation and length of ICU and hospital stay. Inotrope use was similar in both groups. The group receiving opioid anesthesia had an increase in creatinine post operatively which was not observed in the desflurane group. There was no difference in incidence of significant adverse events in either group.

**Conclusions:**

Use of inhalational anesthesia has increased in adult cardiac surgery and has proved to reduce duration of elective ventilation, decrease ICU and hospital LOS, and mortality. Inhalational anesthetics are less well-studied in CCHD. In the current study, desflurane was chosen because of its low solubility, decreased recovery time and lack of metabolism or organ system toxicities. Although it is a popular belief that desflurane is associated with tachycardia and airway irritation, findings of the current study are consistent with those of the previous works demonstrating a lack of these side effects below 1 MAC3. No hemodynamic instability was encountered and there was no evidence that desflurane exerted a negative inotropic effect. Markers of cardio protection were not examined, although desflurane may have had a renal protective effect compared to narcotic technique. In the current study, a desflurane anesthetic for BT shunt decreased the duration of mechanical ventilation and ICU and hospital LOS by nearly three days, with no difference in perioperative morbidity or mortality. Larger studies are required to determine whether these changes result in overall decreased complication rate and morbidity/mortality and whether desflurane has a cardio or renal protective effect in the patient population.

## 1. Background

Repair of cyanotic congenital heart disease often involves the placement of extracardiac systemic-to-pulmonary artery shunts, such as Blalock Taussig (BT), Glenn and Fontan procedures, to allow blood flow to the lungs and thus oxygenation of the body’s tissues.For some patients with severely decreased pulmonary blood flow, a systemic to pulmonary artery shunt is a necessity as a life-saving or temporizing form of palliation (1-5). The modified BT shunt is commonly done in our insitiution utilizing cardiopulmonary bypass. Traditionally these procedures are accomplished by a total intravenous technique (TIVA), with the use of high dose fentanyl, midazolam and a muscle relaxant (6). This technique is used because of its hemodynamic stability, but has a distinct disadvantage in that it increases the period of mechanical ventilation, which predisposes the patient to associated complications and increase intensive care unit (ICU) stay. Additionally, positive pressure ventilation decreases pulmonary blood flow to the surgically created shunts, thereby compromising shunt function. Thus, the shortest possible duration of mechanical ventilation and ICU stay is desirable (1, 5). The use of inhalational anesthetic agents in lieu of narcotics may shorten the duration of mechanical ventilation and ICU stay, but may be associated with myocardial depression and dysarrythmias, especially seen with halothane. Newer inhalational agents, such as sevoflurane and desflurane, are not associated with these cardiac side effects and, due to lower blood solubility, facilitate early awakening and endotracheal extubation (7).

## 2. Objectives

These inhalational agents also provide ‘anestheticpreconditioning’, which protects the heart from ischemic insults frequently encountered during cardiac surgery (8-12). Given its low tissue solubility in addition to low blood solubility, low percentage of metabolism and a lack of effects on other organs at commonly used concentrations, desflurane is particularly attractive as an inhaled agent (9, 12-14). The current study aimed to compare the utility of Desflurane to TIVA in BT shunt procedures for cyanotic congenital cardiac surgery.

## 3. Patients and Methods

Thirty five ASA class III and IV patients between two months and 12 years, scheduled for BT shunt procedures for cyanotic congenital heart disease were studied after receiving approval from local ethical committee and written informed parental consent. Patients requiring emergent intervention or pressor support prior to surgery were excluded.Patients were randomly divided into two groups – TIVA (n = 16) and desflurane (n = 19). Electrocardiogram (ECG), oxygen saturation by pulse oximeter and non-invasive blood pressure were monitored before induction. Institutional practice for all patients undergoing BT shunt is to undergo cardiopulmonary bypass (CPB) following median sternotomy. Patients in both groups did not receive premedication, and induction of anesthesia was achieved with the use of 5-7% sevoflurane in 100% oxygen, 1 mcg/kg fentanyl, 0.05 mg/kg midazolam and 0.1 mg/kg vecuronium. After induction of anesthesia and endotracheal intubation, the technique for maintainence of anesthesia was dictated by sealed envelope randomization.Patients in the TIVA group received an additional 5-10 mcg/kg fentanyl, in incremental doses, as needed to control hemodynamic responses to surgical manipulation, 0.1mg/kg midazolam, 100% oxygen and vecuronium, 0.05mg/kg, as needed for muscle relaxation. In the desflurane group, anesthesia was maintained with desflurane at 0.6 - 1 MAC in 100 % oxygen, 0.05 mg/kg midazolam, 1-2 mcg/kg fentanyl and vecuronium by bolus (0.1 mg/kg), as needed for muscle relaxation/immobility. Pressure control ventilation was commenced in both groups and femoral arterial and central venous catheters were placed.Hydration was accomplished with lactated Ringer’s infusion and blood loss was replaced, keeping hematocrit above 35% in all patients. Hemodynamic parameters were maintained within 20% of baseline values using nitroglycerine (NTG), dopamine and epinephrine infusions at the discretion of the anesthesiologist.At the conclusion of the procedure, both groups were transported to the ICU electively ventilated.On arrival in the ICU, hemodynamic parameters were recorded.Patients in both groups received midazolam intravenously for sedation and paracetamol, 15 mg/kg, intravenously for analgesia.Sedation was stopped once the patients were hemodynamically stable and arousable and the patients were taken off mechanical ventilation using standard weaning protocols. The decision to wean was based on hemodynamic stability and not on the Sedation, Agitation or other systems of sedation scoring. Duration of elective ventilation, inotrope use, ICU and hospital stay were noted. Serum creatinine levels were recorded preoperatively, and then at 48 hours postoperatively. Any serious adverse events, such as stroke, renal failure, arrhythmia, or any other major cardiovascular/neurologic events were recorded. The starting point to note the duration of elective ventilation, inotrope use, ICU and hospital stay commenced from the time the patient arrived in the ICU. SPSS 15.0 for Microsoft Windows was employed to perform statistical analysis. Normally distributed continuous data were compared using the Student t test to compare the two groups. If data points were not normally distributed, Mann-Whitney U tests were used. Categorical variables between the two groups were tested with the chi square/ Fisher exact test. Correlation analysis was carried out to see the relation within each group. Besides , the paired T- test was applied to see the change in variables separately for each group. Repeated measure analysis was carried out to see the trend in heart rate in the intraoperative and postoperative period. A 2-tailed P value < 0.05 was considered statistically significant.

## 4. Results

**Figure 1.**
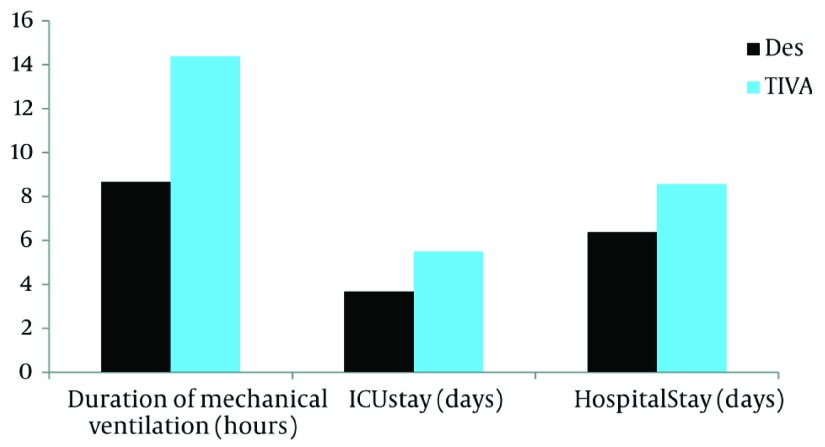
Comparison of Outcome Measures (P < 0.05)

**Figure 2.**
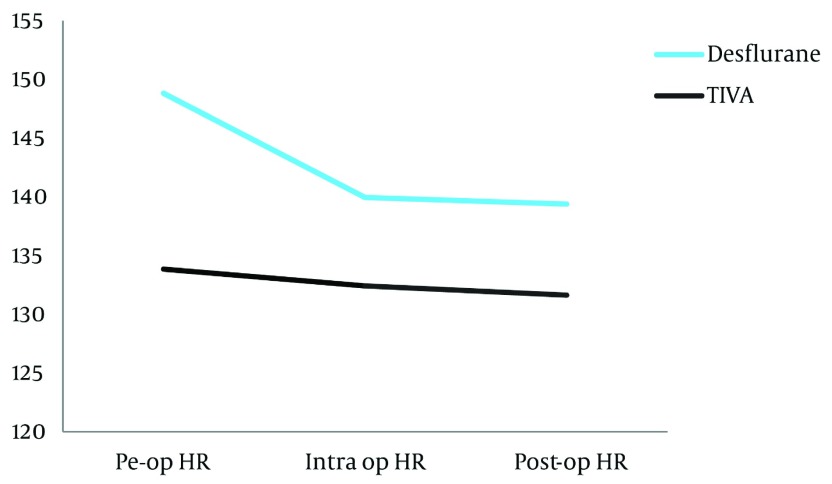
Trend in Heart Rate

The two groups were comparable with regard to the demographic and preoperative data. (Table 1), although heart rate and systolic blood pressure were modestly increased in the TIVA group.The patients of the TIVA group were somewhat older than those of the desfurane group, but the difference was not statistically significant (P=0.053). Statistical analysis and comparing the means of demographic and baseline vital parameters indicated that there was a significant difference in the duration of mechanical ventilation in the two groups (desflurane = 8.66 ± 3.71 hrs; TIVA = 14.37 ± 4.75 hrs, P= 0.001), ICU stay (desflurane = 3.67 ± 1.96 days; TIVA = 5.50 ± 1.63 days, P=0.006) and hospital stay (desflurane = 6.37 ± 2.33 days; TIVA = 8.56 ± 1.41 days, P= 0.002). However there was no significant difference in the duration of inotrope use in the two groups (desflurane = 49.37 ± 32.92 hrs; TIVA = 50.88 ± 19.96 hrs, P=0.367) (Table 2, Figure 1). The creatinine levels measured at 48 hours postoperatively were compared to the preoperative values as paired samples in both groups. There was no significant change in the desflurane group (P = 0.613), but post-op creatinine increased significantly in the TIVA group (P= 0.024). (Table 3).Repeated measure analysis was carried out to see the trend in heart rate from preoperative baseline, during the intraoperative period (measured just before cannulation of the great vessels) and the value on reaching the ICU (Table 4). There was a significant decrease in intraoperative heart rate (within physiological values for age) in the TIVA group (P < 0.05), which then remained relatively constant in the postoperative period. In the desflurane group there was no significant change in heart rate. (Figure 2). Pearson correlation was done between all the variables. There was an association between ICU stay and hospital stay (r = 0.625, P = 0.004). However there was no correlation between the duration of mechanical ventilation and ICU stay (r = 0.011, P = 0.964) and hospital stay (r = 0.144, P = 0.557). There were no serious adverse effects (SAE) in the TIVA group. In the desflurane group one patient had a sudden cardiac arrest within the first 24 hours of surgery and did not survive.The event occurred several hours following surgery and did not appear to be related to either the anesthetic or surgical procedure. Three patients in desflurane group and one in the TIVA group had renal dysfunction in the form of oliguria and deranged creatinine levels. The SAE (death) and renal dysfunction events were not significant when compared using the fisher exact test.

**Table 1.** Statistical Analysis and Comparison in Mean of Demographic and Baseline Vital Parameters

Parameters	Desflurane Group (n = 19)		TIVA Group (n = 16)		P value	Test
	Mean	SD	Mean	SD		
Age, mo	12.53	5.23	16.94	13.95	0.053	Mann-Whitney
Weight, kg	4.83	1.18	4.96	1.75	0.796	Paired t test
Height, cm	62.21	3.59	59.75	4.75	0.091	Paired t test
Heart Rate, beats/min	133.84	14.45	148.81	17.05	0.008	Paired t test
Hematocrit	54.86	8.97	54.83	8.86	0.993	Paired t test
SBP ^a^, mmHg	87.42	7.06	94.19	11.08	0.036	Paired t test
DBP ^a^, mmHg	48.79	7.38	50.81	7.07	0.416	Paired t test
MAP ^a^, mmHg	61.67	5.69	65.27	6.95	0.101	Paired t test
SpO_2, _%	69.37	9.14	69.31	8.316	0.985	Paired t test

^a^Abbreviations: DBP, Diastolic blood pressure; MAP, Mean arterial pressure; SBP, Systolic blood pressure

**Table 2.** Comparison of Outcome Measures

Outcomes	Desflurane Group (n = 19)		TIVA Group (n = 16)		P value	Test
	Mean	SD	Mean	SD		
Duration of mechanical ventilation, h	8.66	3.71	14.37	4.75	0.001	Paired t test
ICU stay, day	3.67	1.96	5.50	1.63	0.006	Paired t test
Hospital stay, day	6.37	2.33	8.56	1.41	0.002	Paired t test
Duration of inotropes use, h	49.37	32.92	50.88	19.96	0.367	Mann-Whitney

**Table 3.** Baseline and 48 HourCreatinine Levels in Both Groups

Creatinine levels	Desflurane Group (n = 19)		P value	Creatinine levels	TIVA Group (n = 16)		P value
	Mean	SD			Mean	Mean	
Preoperative	0.305	0.168	0.613	Preoperative	0.825	0.313	0.024
48 hours post op	0.328	0.377		48 hours post op	1.022	0.437	

**Table 4.** Descriptive Statistics Heart Rate

Parameter	Desflurane group (n = 19)		TIVA group (n = 16)	
	Mean	SD	Mean	SD
Preoperative Heart rate	133.84	14.45	148.81	17.04
Intraoperative Heart rate	132.42	13.33	139.94	13.49
Postoperative Heart rate	131.63	13.41	139.37	10.43

## 5. Discussion

Cyanotic congenital heart disease often requires placement of systemic to pulmonary artery (PA) shunts to allow blood flow to the lungs and thus oxygenation of the body’s tissues. Palliative surgery increases pulmonary blood flow, temporarily stabilizing the patient and allowing him/her to grow and undergo future corrective or palliative procedures. The most common shunt is the BT shunt, named after Blalock and Taussig who first described it in 1945 (2). Here, the shunt is from a systemic artery (e.g., the subclavian or the aorta) to the pulmonary artery (main, right, or left).

Another type of palliative shunt is from a systemic vein (superior vena cava [SVC]) to the PA.This is termed a Glenn shunt. Because this shunt directs only deoxygenated blood to the PA prior to its entry into the heart, it reduces the volume load on the heart. Because the driving pressure in the Glenn shunt is the SVC pressure (rather than right ventricular), the PA pressures must be low for this to be successful.PA pressures typically decrease to acceptable levels to allow proper function of a Glenn shunt after an infant is older than 2-3 months. Palliative shunts have become less common treatments for many cyanotic heart malformations since the advent of early corrective procedures.Nevertheless, certain subsets of patients require initial palliative surgery because their cardiac disorders are not generally amenable to initial corrective surgery, owing to either anatomic or physiologic causes.Moreover it may be an institutional practice in some centers to delay definitive corrective procedures until 12-18 months of age. In these patients a systemic to pulmonary artery shunt remains a necessity as a life-saving or temporizing form of palliation (1, 4, 5). The modified BT shunt involves interposing a Gore-Tex graft between the subclavian or innominate artery and the ipsilateral PA (3). This shunt can be performed with or without CPB via a thoracotomy or median sternotomy. An IV induction with ketamine or fentanyl is desirable, but most infants and children will tolerate a mask induction with either sevoflurane or halothane as there is a parallel decrease in pulmonary vascular resistance (PVR) and systemic vascular resistance (SVR). Maintenance of anaesthesia with high dose fentanyl, a muscle relaxant, and a benzodiazepine is most commonly done because of the hemodynamic stability achieved.Opioids provide profound analgesia, attenuation of unwanted visceral responses to surgery, and, in high doses, ablation of stress responses (6, 15). Duncan et al. compared different doses of fentanyl (range 2-150 µg/kg) in infants and young children undergoing cardiac surgery. They found that fentanyl 2 µg/kg failed to prevent significant rises in cortisol and norepinephrine, whereas 25 and 50 µg/kg were as effective as higher doses but provided greater cardiovascular stability (16). The opioid technique, however, has the disadvantages of bradycardia, oversedation and delay in weaning from postoperative mechanical ventilation (17, 18) which not only predisposes the patient to complications of prolonged mechanical ventilation and ICU stay, but, due to positive pressure ventilation, also causes lower pulmonary blood flow to the surgically created shunts, thereby compromising shunt function.Since it is desirable to decrease duration of mechanical ventilation and reduce ICU stay, the use of volatile anesthetic agents may be beneficial in terms of shortened duration of mechanical ventilation and ICU stay. Volatile anesthetic agents when compared with TIVA/opioid-based technique in CABG surgery with or without bypass, were found to decrease the time of mechanical ventilation, shorten ICU stay and reduce risk of myocardial infarction (7). Some volatile agents, however, are associated with myocardial depression and dysarrythmias, especially halothane. These negative inotropic effects are significantly less potent in the newer inhalational agents, sevoflurane and desflurane (19). Moreover both sevoflurane and desflurane have been found to have cardioprotective effects which are attributed to ‘anesthetic preconditioning’ that protects the heart from ischemic insult frequently encountered during cardiac surgery.The first clinical trial to investigate the clinical efficacy of volatile anesthetics in CABG was in 2002, reporting that sevoflurane preserved global hemodynamic and left ventricular function with a lower postoperative troponin I compared with total intravenous anesthesia (8). Since then, well designed animal and human studies (in the adult population) have repeatedly demonstrated exposure of the myocardium to a volatile anesthetic before a period of ischemia significantly protects the myocardium against subsequent ischemia- reperfusion injury (9-11). Both sevoflurane and desflurane are reported to have cardioprotective effects (12). In one study desflurane conferred a greater degree of cardiac protection than sevoflurane (14). Desflurane has the advantage of having lower blood gas and tissue solubility than other volatile agents,allowing for rapid decrease of alveolar concentrations during elimination, thereby facilitating early awakening and extubation and therefore reduced duration of positive pressure ventilation. Thus desflurane may have an advantage over sevoflurane in fast tracking, particularly following longer cases, such as cardiac surgery. Desflurane has also shown to have cardioprotective effect in terms of ICU stay and weaning from mechanical ventilation (9). Finally, absence of metabolic or breakdown byproducts, such as fluoride or Compound A in desflurane, make it an attractive option in cardiac surgery, wherein CPB may have a deleterious effect on the renal system. A thorough literature search was done but only a few studies comparing inhalational agents to TIVA in cardiac surgery patients were found. All these studies were in the adult population who underwent CABG surgery. There is no study wherein these two techniques are compared for maintenance of anaesthesia in cyanotic children undergoing palliative shunt surgery. Fabio Guarracino and Giovanni Landoni evaluated the effects of volatile anesthesia versus total intravenous anesthesia on cardiac troponin release in off-pump coronary artery bypass grafting (OPCAB) patients and found that myocardial damage could be reduced by volatile anesthetics during OPCAB (11). In another meta-analysis, the authors examined studies comparing a total intravenous anesthesia regimen versus an anesthesia plan including desflurane or sevoflurane. They concluded that desflurane and sevoflurane have cardioprotective effects that result in decreased morbidity and mortality (12). Desflurane is an acceptable volatile agent in children undergoing non-cardiac surgery. Desflurane administered at 1 MAC or less does not adversely affect hemodynamic parameters, hepatic or renal function in children. Desflurane may be preferred over sevoflurane when early recovery from anaesthesia is warranted (13). Similar results were found in the patients under study, wherein there was a significant decrease in the duration of mechanical ventilation, ICU stay, and the total duration of stay in the hospital in the desfluranegroup. Additionally, in the current study TIVA was associated with an increase in post-operative creatinine level which was not observed in the desflurane group. It is unclear whether desflurane may have a renal protective quality that has not been well-studied.CPB is known to have deleterious effects on renal function and like many other institutions, we routinely measure post-operative indices of renal function to better address CPB-associated renal insult (20). A potential renal protective property of desflurane following CPB is an intriguing possibility that merits further study. Desflurane is known to have negative inotropic effects (19). In the current study there was no difference in the duration of inotrope use in the two groups. There is evidence that desflurane confers myocardial protection in adult patients undergoing cardiac surgery (11, 12, 14), but as the current study did not measure ischemic markers, it is unable to comment on this. There were no significant serious adverse events in either group that could be attributed to choice of anaesthesia technique. The use of desflurane is associated with increased incidence of agitation especially in the pediatric age group, but no patient in the current study had any episode of agitation / seizure. This may be because fentanyl 1-2 mcg/kg body weight was used during induction. Fentanyl has been found to prevent emergence agitation while preserving the rapid recovery associated with desflurane anesthesia in children undergoing non cardiac surgery (21). Additionally, the patients under study were not awakened until achieving hemodynamic stability in the ICU and this extended time to awakening likely obviated any emergence delirium. Desflurane is also associated with sympathetic stimulation when administered in concentrations above 1.5 MAC.There was a significant decrease in heart rate (within physiological values for age) in the TIVA group which was not observed in the desflurane group. The desflurane patients had a relatively stable heart rate. The current study results have been favorable for faster recovery in the desflurane group, but the study had some limitations. First, it was a single center trial and was not blinded. Second, since ischemic markers were not studied, objective analysis of myocardial damage was not done. Hemodynamic parameters, such as arterial blood pressure, central venous pressure and central venous oximetry, which change frequently during surgery, were not recorded at the three time intervals when heart rate was recorded. TIVA with fentanyl, midazolam and non-depolarizing muscle relaxant, is an established and accepted technique for BT shunt and other cardiac surgeries, but has disadvantages of prolonged mechanical ventilation, potential shunt compromise secondary to positive pressure ventilation, and prolonged ICU stay. The current study demonstrated that the use of desflurane along with low dose fentanyl is a favorable alternative to the high dose fentanyl, and benzodiazepine combination. Although the patients under study were managed by an intensivist, independent of the anesthesia team, and weaned according to institutional practice, the study was not blinded and it is not possible to completely rule out bias or Hawthorne effect. It has been previously demonstrated that early postoperative extubation correlates with decreased invasive intervention and use of inotropes in demographically matched groups of like hemodynamic stability (22). A patient`s being awake and breathing spontaneously versus unconscious and on a ventilator, may influence therapeutic strategy. Even a completely blinded prospective study, thus, may not eliminate this limitation.Although the current study did not directly measure markers of cardiac or renal injury following CPB, the lack of a post-operative increase in creatinine in the desflurane group is of interest and possible cardioprotective and renal protective effects of desflurane in the pediatric population should be further studied.
